# Deleting an xylosidase-encoding gene *VdxyL3* increases growth and pathogenicity of *Verticillium dahlia*

**DOI:** 10.3389/fmicb.2024.1428780

**Published:** 2024-07-22

**Authors:** Yongtai Li, Shenglong Song, Bin Chen, Yong Zhang, Tiange Sun, Xiaohu Ma, Yanjun Li, Jie Sun, Xinyu Zhang

**Affiliations:** ^1^The Key Laboratory of Oasis Eco-agriculture, Agriculture College, Shihezi University, Shihezi, Xinjiang, China; ^2^Key Laboratory of Prevention and Control of Invasive Alien Species in Agriculture and Forestry of the North-western Desert Oasis (Co-construction by Ministry and Province), Ministry of Agriculture and Rural Affairs, Ürümqi, Xinjiang, China

**Keywords:** cotton, RNA-sequencing, xylosidase, *VdxyL3*, *Verticillium dahliae*

## Abstract

**Introduction:**

*Verticillium dahliae* causes a devastating Verticillium wilt disease on hundreds of plant species worldwide, including cotton. Understanding the interaction mechanism between *V. dahliae* and its hosts is the prerequisite for developing effective strategies for disease prevention.

**Methods:**

Here, based on the previous observation of an xylosidase-encoding gene (*VdxyL3*) in *V. dahliae* being obviously up-regulated after sensing root exudates from a cotton variety susceptible to this pathogen, we investigated the function of *VdxyL3* in the growth and pathogenesis of *V. dahliae* by generating its deletion-mutant strains (*ΔVdxyL3*).

**Results:**

Deleting *VdxyL3* led to increased colony expansion rate, conidial production, mycelial growth, carbon and nitrogen utilization capacities, and enhanced stress tolerance and pathogenicity of *V. dahliae*. *VdxyL3* is a secretory protein; however, *VdxyL3* failed to induce cell death in *N. benthamiana* based on transient expression experiment. Transcriptomic analysis identified 1300 genes differentially expressed (DEGs) between wild-type (Vd952) and *ΔVdxyL3* during infection, including 348 DEGs encoding secretory proteins, among which contained 122 classical secreted proteins and 226 non-classical secreted proteins. It was notable that of the 122 classical secretory proteins, 50 were carbohydrate-active enzymes (CAZymes) and 58 were small cysteine rich proteins (SCRPs), which were required for the pathogenicity of *V. dahliae*.

**Conclusion:**

The RNA-seq data thus potentially connected the genes encoding these proteins to the pathogenesis of *V. dahliae*. This study provides an experimental basis for further studies on the interaction between *V. dahliae* and cotton and the pathogenic mechanism of the fungus.

## 1 Introduction

Cotton is an extremely important economic crop in the world, providing 35% of all natural fibers for the textile industry, as well as a source of edible oil and animal feed (Billah et al., 2021; Man et al., 2022). Verticillium wilt, caused by *Verticillium dahliae*, is a vascular infection disease that severely affects cotton production worldwide and is known as cotton cancer (Aguado et al., 2008; Su et al., 2018). Microsclerotia serves as a structure for *V. dahliae* that infects hosts. The structure can help the pathogen tolerate various environmental stresses, such as high temperature, cold, drought and flood, and can survive in the soil environment for at least 14 years (Short et al., 2015). When the soil environment is suitable, the microsclerotia can produce mycelium, which invades plants through root tip, reaches vascular tissues and proliferates, leading to characteristic wilt symptoms, including leaf yellowing, wilting, necrosis, vascular discoloration and even plant death (Fradin and Thomma, 2006; Tian and Kong, 2022). Few effective strategies are available for the consistent management of Verticillium wilt due to the complex of pathogenic mechanism of *V. dahliae*.

During the invasion process, pathogen produces cell wall degrading enzymes (CWDEs) that can degrade the cell wall polysaccharides of host plants to achieve invasion and colonization (Nuhse, 2012; Kubicek et al., 2014). Previous studies have shown that many CWDEs are virulence factors and associated with pathogen infection processes (Morales-Cruz et al., 2015; Quoc and Chau, 2017). The plant cell wall comprises cellulose, hemicellulose and pectins. Xylan is the main hemicellulose component that are widely present in the cell walls of various types of plants and is the second most abundant polysaccharide in terms of biomass (Bastawde, 1992). Xylan is made from units of xylose and contains predominantly β-D-xylose units linked by β-1,4 glycosidic bonds (Bhardwaj et al., 2019). Xylan is degraded mainly by backbone-degrading enzymes xylanase and xylosidase. Many genes encoding xylanase in fungi have been found to be required for virulence. The effects of targeted disruption of some individual xylanase genes support their direct involvement in infection and pathogenicity, such as *Xyn11* in *Botrytis cinerea* (Brito et al., 2006), *SsXyl1* in *Sclerotinia sclerotiorum* (Yu et al., 2016), *VmXyl1* in *Valsa* mali (Yu et al., 2018), and *ppxyn1* and *ppxyn2* in *Phytophthora parasitica* (Lai and Liou, 2018). As another key enzyme for degrading xylan, however, the role of xylosidase in fungal pathogenesis has not been reported yet.

Classical models of plant-pathogen interactions propose that plants utilize two key defense mechanisms when responding to pathogen invasion. The initial defense mechanism recognizes pathogenic microbe-associated molecular patterns (MAMPs) or pathogen-associated molecular patterns (PAMPs) via pattern recognition receptors (PRRs) situated on the cell membrane surface (Akira et al., 2006; Escocard de Azevedo Manhães et al., 2021). This process initiates a PAMP-induced immune (PTI) anti-disease response. Effector-induced immunity (ETI) in plant cells is facilitated by nucleotide-binding/leucine-rich repeat (NB-LRR)-containing receptors, which constitute the second layer of the immune system (Hein et al., 2009; Zhou and Zhang, 2020; Paluchowska et al., 2022). In addition to the virulence effect mentioned above, some CWDEs can also act as PAMPs, which can induce plant immune responses, such as *VdGAL4*, *VdEG1*, *VdEG3*, *VdPEL1* and *VdCUT11* (Gui et al., 2017, 2018; Yang et al., 2018b; Wen et al., 2023). A few xylan-degrading enzymes were reported to be PAMPs, such as *PsXEG1* in *Phytophthora sojae* (Ma et al., 2015), *BcXYG1* and *BcXYl1* in *Botrytis cinerea* (Zhu et al., 2017; Yang et al., 2018a), and *VdEIX3* in *V. dahliae* (Yin et al., 2021).

Our previous RNA-seq results revealed that many CWDE genes, including an xylosidase encoding gene *VdxyL3*, were obviously up-regulated after sensing root exudates from cotton variety susceptible to *V. dahliae* (Zhang et al., 2020), suggesting that they may play important roles in the pathogenic process of *V. dahliae*. The main objectives of the current study were to: (1) investigate the function of *VdxyL3* in growth and virulence of *V. dahliae*; (2) determine whether *VdxyL3* is a secreted protein and investigate whether it can act as a PAMP triggering cell death response in *N. benthamiana*; (3) determine whether the deletion of *VdxyL3* affects the expression of other genes in *V. dahliae* and if so, what they are. The results generated in this study provide an experimental basis for further studies on the interaction between *V. dahliae* and cotton and the pathogenic mechanism of the fungus.

## 2 Materials and methods

### 2.1 Fungal strain, plant material, and culture conditions

The wild-type *V. dahliae* strain Vd592 is a highly pathogenic and defoliating strain. The fungus was maintained on potato dextrose agar (PDA) medium at 25°C in the dark. Upland cotton cultivar ‘Xinluzao 7’ and ‘Zhong Zhimian 2’ susceptible to *V. dahliae* and Sea Island cotton cultivar ‘Hai 7124’ resistant to *V. dahliae* were used in this study. Cotton seeds were grown in a plant incubator at 28°C with a photoperiod of 16h light/8h dark and a relative humidity of 60%. *N. benthamiana* seeds were grown in a greenhouse grown at 25°C for 6 weeks under a photoperiod of 16h light/8h dark and a relative humidity of 60%.

### 2.2 DNA, RNA extraction, and cDNA synthesis

Fungal DNA was extracted using the Fungal DNA kit (Omega Inc., USA). Total RNA of *V. dahliae* was extracted using the Fungal RNA kit (Omega Inc., USA). Total RNA of cotton tissues was extracted using the EASYspinPlus Plant RNA Extraction Kit (Aidlab, Beijing, China). After removing genomic DNA using DNase I, the integrity of total RNA was assessed by 1.0% agarose gel electrophoresis, and RNA concentration was determined by NanoDrop 2000. The first strand of cDNA was synthesized using EasyScript One-Step gDNA Removal and cDNA Synthesis Super Mix EasyScript kit (TransGen, Beijing, China) following the manufacturer’s instructions and stored in a −20°C freezer.

### 2.3 Bioinformatics analysis

The GFF3, CDS (Coding sequence), genomic and protein sequence files of *V. dahliae* ASM15067v2 were downloaded from NCBI.^1^ All xylosidase genes based on annotation were preliminarily searched in GFF3 file using TBtools software. The glycoside hydrolase domain in xylosidases was predicted using the online SMART software.^2^ The transmembrane predictor characteristics of the *VdxyL3* protein were predicted by the online software TMHMM.^3^ The potential signal peptide of *VdxyL3* was predicted using SignalP 5.0.^4^ The phylogenetic analysis was performed by the MEGA 11.0 software using maximum-likelihood method.

### 2.4 Target gene knockout and complementation

The pGKO_2_-Gate and pSULPH-mut-RG#PB vectors used in this study were kindly donated by Dr Zhaosheng Kong, Institute of Microbiology, Chinese Academy of Sciences. To construct a deletion vector, *VdxyL3* was targeted by replacing its target fragment (2544 bp) with a hygromycin resistance gene (*HPH*) fragment (1908 bp), which was amplified from pUC-*HPH* plasmid DNA using *HPH*-F/*HPH*-R primers. Paired primers *VdxyL3*-Flank-5F/*VdxyL3*-Flank-5R and *VdxyL3*-Flank-3F/ *VdxyL3*-Flank-3R were used to amplify the upstream (1032 bp) and downstream (1000 bp) flanking fragments of the target fragment using Vd592 genomic DNA as template (Supplementary Table 1). All PCR products (1032, 1908, and 1000 bp) were then integrated into pGKO_2_-Gate vector via homologous recombination using the ClonExpress II one-step cloning kit (Vazyme Biotech Co. Ltd, Nanjing, China) according to the manufacturer’s instructions. The resultant vector was transformed into Vd592 by the PEG-mediated transformation method (Wang et al., 2013). The correct transformants were screened on PDA containing suitable antibiotics and confirmed by PCR using the primers Test-*VdxyL3*-F1/R1, Test-*VdxyL3*-F2/R2 and Test-*VdxyL3*-F3/R3 (Supplementary Table 1).

To construct a complementary vector, a fragment about 4.7 kb in size including promoter, coding region and terminator sequences of *VdxyL3* was amplified using Vd592 genomic DNA as template and Promoter-F/*VdxyL3*-R as primers. The PCR product was integrated into the pSULPH-mut-RG#PB vector using the ClonExpress II one-step cloning kit. The resultant vector was transformed into Vd592 by the PEG-mediated transformation method (Wang et al., 2013). The correct transformants were confirmed by PCR with primers Test-*Hyg*-F/R (Supplementary Table 1).

### 2.5 Fungal growth

The wild type (Vd592), deletion and complementary mutants were inoculated into liquid CM medium (Complete medium) and incubated at 25°C for a week. Conidia from different strains were collected, and were diluted to 1 × 10^7^CFU/mL with water. Then 10 μL of conidial suspension was dropped onto PDA, CM and Czapek Dox medium and incubated at 25°C. The colony diameter was measured and photographed at 15 days post inoculation. The conidial concentration of each strain was adjusted to 1 × 10^7^CFU/mL using a hemacytometer and was then inoculated into liquid CM medium cultured on a shaker (180 rpm/min) at 25°C. The conidial yield was determined using a blood cell counting plate under a microscope every day. Conidia from different strains were diluted to 1 × 10^3^ CFU/mL and were then evenly distributed on PDA medium using a triangular spreading rod. After 24h of incubation, the monoconidial growth was observed under a microscope. Each strain was replicated at least three times.

### 2.6 Stress response assays

To test the sensitivity of different strains to abiotic stresses, 10 μL of conidial suspension (1 × 10^7^ CFU/mL) was incubated on PDA solid medium containing 1 M sorbitol, 0.02% Congo red (CR), 30% H_2_O_2_, 30 μg/mL calcofuor white (CFW), 1 M NaCl or 0.002% SDS. The colony diameters of all strains were measured, and the colony morphology was observed and photographed after 15 days of incubation. The formula of relative growth inhibition rate was calculated as follows: relative growth inhibition rate = (CK colony diameter – treatment colony diameter)/CK colony diameter × 100% (Liu et al., 2017). Each strain was replicated at least three times.

### 2.7 Carbon and nitrogen sources utilization assays

To examine the carbon utilization capacity of different strains, glucose (10g/L), xylan (10g/L), D-xylose (10g/L), pectin (10g/L) or cellulose (10g/L) was added individually into Czapek Dox media without carbon sources (Wang et al., 2021). Carbon-free Czapek Dox media were used as controls. Drops of conidial suspension (1 × 10^7^ CFU/mL) were inoculated on Czapek Dox media with or without carbon sources and incubated at 25°C. For nitrogen utilization assays, tryptophan (10g/L), valine (10g/L), leucine (10g/L), serine (10g/L) or histidine (10g/L) was added individually into Czapek Dox media without nitrogen sources (Júnior et al., 2009). Nitrogen source-free Czapek Dox media were used as controls. Drops of conidial suspension (1 × 10^7^ CFU/mL) were inoculated on Czapek Dox media with or without nitrogen sources and incubated at 25°C. The colony diameters of all strains were measured after 15 days of inoculation. Each strain was replicated at least three times.

### 2.8 Pathogenicity and mycelial penetration assays

Pathogenicity assays were carried out on cotton seedlings using a root irrigation method as previously described (Xiong et al., 2020). Briefly, cotton seedlings at two-true-leaf stage were infected with conidial suspension (1 × 10^7^ CFU/mL) of different strains (Vd592, Δ*VdxyL3* and Δ*VdxyL3-C* strains). Verticillium wilt disease symptoms, vascular discoloration and disease index were assessed at 14 and 21 dpi (days post inoculation). Infected plants were graded on a scale of zero to four based on the symptoms observed in their cotyledons and new leaves (Wang et al., 2021). The Disease Index (DI) was calculated using the following equation: DI = [(Σ disease grade × number of infected plants)/ (total number of sampled plants × 4)] × 100 (Wang et al., 2021; Chen et al., 2023). For fungal biomass quantification, roots and stems of three plants were harvested at 14 and 21 dpi and used for genomic DNA extraction. The fungal biomass was quantified by qRT-PCR with specific primers ITS1-F and ST-Ve1-R. The cotton *GhUBQ7* gene was used as the endogenous plant control (Supplementary Table 1). For the fungal recovery assay, cotton stem was collected at 21 dpi (day post inoculation) and cut into segments of 2–3 cm. The stem segments were soaked in 3% NaClO for 2 min, and then washed with distilled water at least three times. Then the stem segments were placed onto PDA medium and incubated at 25°C for 7 days to observe the fungal colonies.

To investigate the capacity of penetration through cellophane of different strains, drops of conidial suspension (1 × 10^7^ CFU/mL) of each strain were inoculated and cultured on PDA medium overlain with cellophane membranes for 7d and photographed, after removing the cellophane membranes the plates were further incubated for another 7d, and the colony size was then measured and photographed. All experiments were repeated three times.

### 2.9 Yeast signal sequence trap system

A yeast signal sequence Trap System was used to validate the function of the predicted signal peptide of the *VdxyL3* gene as described previously (Wang et al., 2022). The region encoding the signal peptide of *VdxyL3* was amplified using specific primers F/R (Supplementary Table 1) and integrated into the pSUC2 vector to generate pSUC2-*VdxyL3*^sp^. The empty pSUC2 and pSUC2-*Avr1b*^sp^ vectors were used as negative and positive controls, respectively. The pSUC2-*VdxyL3*^sp^ vector was then transformed into the yeast strain YTK12 and screened on CMD-W (lacking tryptophan) medium (SS/-Trp with Agar medium). The positive colonies were incubated on YPRAA medium containing 2% raffinose. In addition, the reduction of 2,3,5-triphenyltetrazolium chloride (TTC) stained to red insoluble 1,3,5-triphenylformazan (TPF) was used to detect the sucrose invertase activity of SP. Transformed yeast strains were cultured in CMD-W medium (30°C, 24h), harvested and stained with 2% TTC (30°C, 30min). Invertase activity was determined by observing the changes of TTC color.

### 2.10 *Agrobacterium* infiltration assays

The coding sequence of *VdxyL3*, including the signal peptide sequence was amplified from cDNA of Vd592, inserted into the PYBA-1132 vector and transformed into *A. tumefaciens* GV3101 through heat shock (Supplementary Table 1). For transient expression in *N. benthamiana*, the *A. tumefaciens* GV3101 strain carrying PYBA-1132 vector was resuspended in MES buffer (10mM MgCl_2_, 10mM MES, 10 μM acetosyringone and kept in dark for 2 to 3h before infiltration) as previously reported (Yang et al., 2018b). The suspended *A. tumefaciens* cells with an optical density at 600 nm of 0.8 were then injected into 4-week-old *N. benthamina* leaves. Cell death was examined and photographed at 7 d post injection. Negative and positive controls were set by using *A. tumefaciens* GV3101 carrying PYBA-1132 and PYBA-1132:BAX vectors, respectively. To examine the suppression of cell death induction in *N. benthamiana*, *A. tumefaciens* GV3101 carrying PYBA-1132:*VdxyL3* and PYBA-1132:BAX were mixed in a 1:1 ratio and the mixed strains were then used in infiltration of *N. benthamiana* leaves.

### 2.11 RNA-sequencing (RNA-seq)

Cotton seedlings at two-true-leaf stage were infected with conidial suspension (1 × 10^7^CFU/mL) of wild-type (Vd592) and *VdxyL3* deletion mutant. The roots were collected at 36h and 3d post infection and used for RNA-seq. Total RNA extraction and quality assessment, cDNA library preparation, data assembly, sequence alignment to reference genomes, and unigene annotation were performed by Biomarker Technologies Co (Beijing, China). Total RNA was extracted from cotton roots using RNAprep Pure Plant Kit (Tiangen, Beijing, China). Briefly, the cDNA libraries were sequenced using an illumina novaX plus platform that generates paired-end reads with a length of 150bp. Raw sequencing data were processed using the BMKCloud online platform^5^ to remove low-quality sequences and unreliable poly-N sequences. The clean reads were aligned to the *V. dahliae* reference genome (VdLs.17) assembly with HISAT2 tool. Stringtie was used to reconstruct the transcripts and calculate the expression of all genes in each sample, presented as FPKM value.

### 2.12 Differentially expressed genes (DEGs) and gene annotation analysis

Differential analysis was performed using edgeR software with the screening criteria of fold change ≥ 1.5 and *P*-value < 0.05. Gene Ontology (GO) enrichment analysis and Kyoto Encyclopedia of Genes and Genomes (KEGG) pathway enrichment were performed using the online Microbiology Letter Mapping^6^ analysis. In addition, secreted proteins were predicted based on SignalP 5.0, TMHMM 2.0, and SecretomeP 2.0 (Krogh et al., 2001; Bendtsen et al., 2004; Petersen et al., 2011). The prediction of PHI homologs was performed based on the PHI database (Liu et al., 2022). Annotation of putative CAZymes was performed using a Hidden Markov Model (HMM) routine based on the Carbohydrate-Activity-enzymes database (Lombard et al., 2014).

### 2.13 Gene expression validation

The RNA-seq data were confirmed by selecting 12 DEGs and measuring their expression levels by qRT-PCR. The cDNAs were synthesized from total RNA used for RNA-seq using M-MLV. The PCR reactions were performed in a total volume of 20 μL including 10 μL 2 × ChamQ universal SYBR qPCR master mix, 0.4 μL primer-F, 0.4 μL primer-R, 2 μL (100 ng) cDNA, and 7.2 μL ddH_2_O. The qRT-PCR program included an initial denaturation step at 95°C for 30s, followed by 40 cycles of 10s at 95°C and 30 s at 60°C. The expression levels were normalized to the expression of the reference gene *β-tubulin* (VDAG_10074). The relative expression levels of the genes were calculated with the formula 2^–ΔΔ^ ^Ct^.

### 2.14 Statistical analysis

For each experiment, three independently repeated experiments were carried out. Statistical analyses were performed using SPSS statistical software package (v26.0). One-way analysis of variance (ANOVA) was applied and followed by the Student-Newman-Keuls (SNK) test to determine significant differences between treatments at p value of 0.05 or 0.01.

## 3 Results

### 3.1 Bioinformatics and expression analysis of VdxyL genes

**FIGURE 1:**
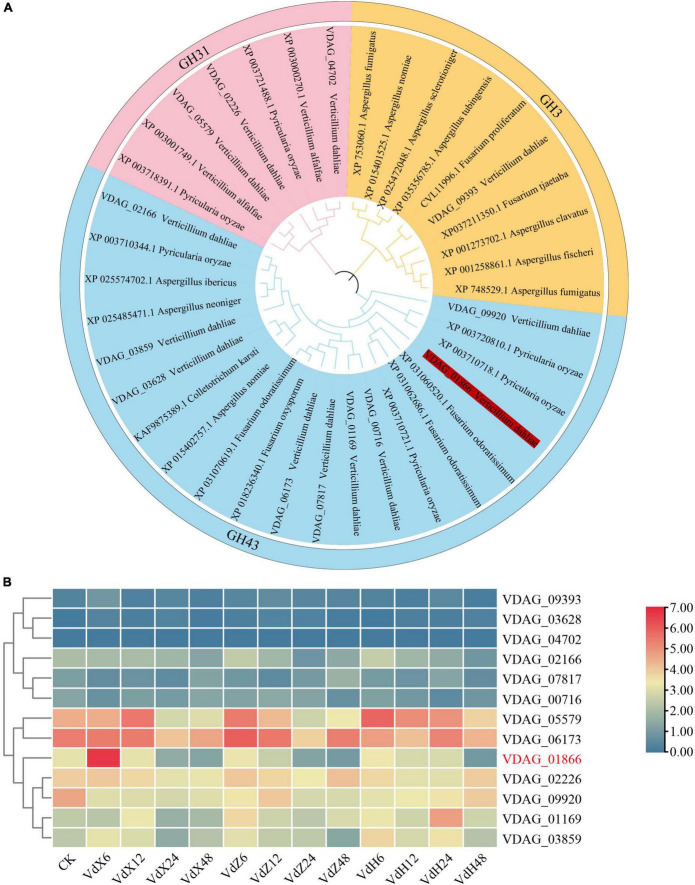
Phylogenetic tree and expression profiles of *VdxyL* genes. **(A)** The phylogenetic tree of xylosidase genes from *V. dahliae*, other fungi (*Pyricularia oryzae, Verticillium alfalfa, Aspergillus fumigatus, Aspergillus nomiae, Aspergillus sclerotioniger, Aspergillus tubingensis, Fusarium proliferatum, Fusarium tjaetaba, Aspergillus clavatus, Aspergillus fischeri, Fusarium odoratissimum, Fusarium oxysporum, Colletotrichum karsti, Aspergillus neoniger, Aspergillus ibericus*). The phylogenetic tree was constructed by MEGA 11.0 using the maximum likelihood method with 1,000 bootstrap tests. Five subfamilies of glycoside hydrolases were distinguished by five different colors. The *VdxyL3* gene selected for further study is highlighted in red. **(B)** The expression profiles of the 13 *VdxyL* genes in *V. dahliae* after sensing cotton root exudates. VdX6, VdX12, VdX24 and VdX48 represent *V. dahliae* samples cultured with root exudates from cotton variety (Xinluzao 7) susceptible to *V. dahliae* for 6, 12, 24, and 48 h, respectively. VdZ and VdH represent *V. dahliae* samples cultured with root exudates from the cotton variety resistant to *V. dahliae*, Zhongzhimian 2 and Hai7124, respectively. CK represents *V. dahliae* culture without root exudate. The color of the scale, from blue to red, represents low to high expression. The deleted gene was highlighted by red color.

A total of 13 xylosidase-encoding genes were identified in the *V. dahliae* genome. The 13 *V. dahliae* xylosidase genes, together with 25 xylosidase genes from other fungi and bacteria were used to generate a phylogenetic tree. The results showed that the 38 *VdxyL* genes were classified into three subfamilies (GH31, GH3 and GH43) (Figure 1A).

Based on our previous RNA-seq results (Zhang et al., 2020), a heat map was generated for the 13 *VdxyL* genes to investigate the expression profiles of these genes after sensing root exudates from cotton varieties resistant or susceptible to *V. dahliae*. Among the 13 *VdxyL* genes, only VDAG_01866 (*VdxyL3*) of the GH43 subfamily showed obvious up-regulation in response to the root exudate (VdX6) from the susceptible cotton variety (Xinluzao 7) and no significant change in response to root exudates (VdH and VdZ) from the resistant cotton varieties (Hai7124 and Zhongzhimian 2). The remaining 12 *VdxyL* genes had no significant difference in terms of their responses to root exudates from the three cotton varieties. These results suggested that *VdxyL3* may play an important role in the pathogenicity of *V. dahliae* (Figure 1B).

### 3.2 Deletion of *VdxyL3* leads to increased fungal growth

**FIGURE 2:**
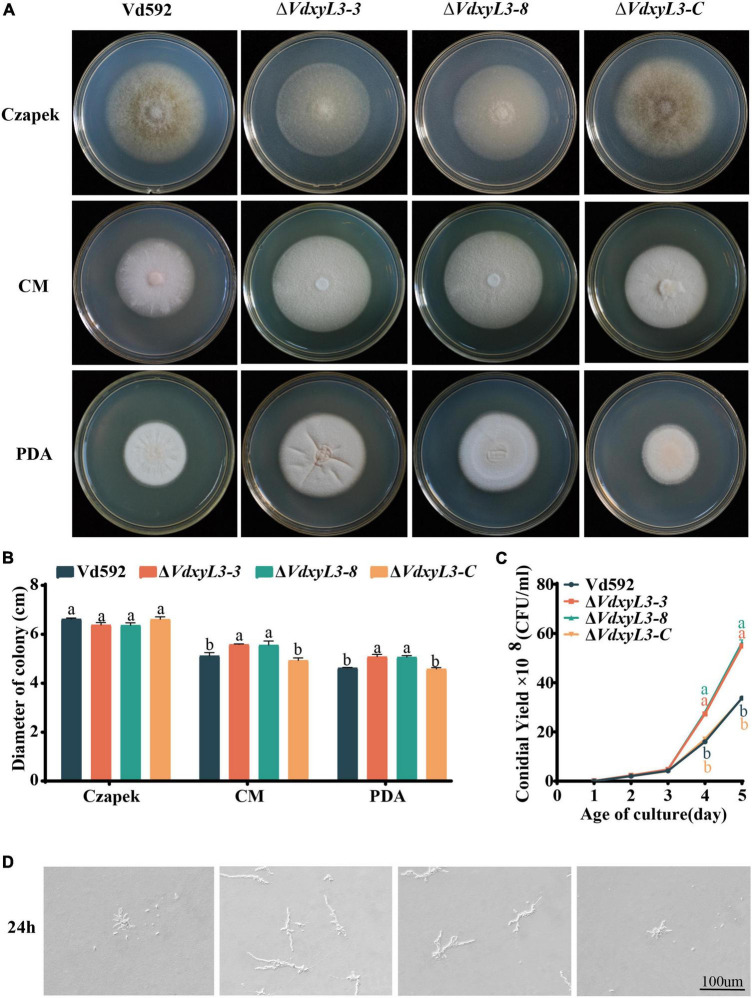
Colony morphology, conidial yield and monoconidial germination of different *V. dahliae* strains. **(A)** Colony morphology of different *V. dahliae* strains at 15 days post incubation on Czapek Dox, CM and PDA media. **(B)** Colony diameter statistics of different *V. dahliae* strains at 15 days post incubation on Czapek Dox, CM and PDA media. Error bars were based on three independent experiments. **(C)** Number of conidia produced by different *V. dahliae* strains in Czapek Dox medium at 25°C. The number of conidia was determined at 1, 2, 3, 4, 5 days post incubation, respectively. Error bars were based on three independent experiments. **(D)** Monoconidial growth on PDA medium at 24 h post incubation. Data were statistically analyzed using IBM SPSS Statistics 26.0. Significant differences between treatments were analyzed by one-way ANOVA using the Student-Newman-Keuls (SNK) test (different letters on error bars indicate significant difference at *P* ≤ 0.05).

To determine the biological function of *VdxyL3*, we generated *VdxyL3* deletion mutants and complementation strain via homologous recombination mediated by a PEG-mediated transformation method. Two independent deletion *V. dahliae* mutants (*ΔVdxyL3-3* and *ΔVdxyL3-8*) and one complementation *V. dahliae* mutant (Δ*VdxyL3-C*) were obtained. After verifying by PCR and qRT-PCR (Supplementary Figures 1A–C), they were used in further investigation. Although there was no significant difference in colony size between the Δ*VdxyL3* mutants and Wild Type (Vd592) on Czapek Dox medium, the colony size of the two independent Δ*VdxyL3* mutants was obviously larger than that of Vd592 and Δ*VdxyL3-C* strains on both PDA and CM media (Figures 2A, B). The conidial yield of the Δ*VdxyL3* mutants were significantly higher than that of the Vd592 and Δ*VdxyL3-C* strains (Figure 2C). The monoconidial growth investigation showed that the two Δ*VdxyL3* mutants produced more and longer mycelium at 24h after inoculation on PDA medium (Figure 2D). These results showed that the deletion mutants grew faster than Vd592, suggesting that deletion of *VdxyL3* leads to increased fungal growth, including colony expansion, increased conidial production and mycelial growth.

### 3.3 Deletion of *VdxyL3* leads to enhanced tolerance of *V. dahliae* to different stresses

**FIGURE 3:**
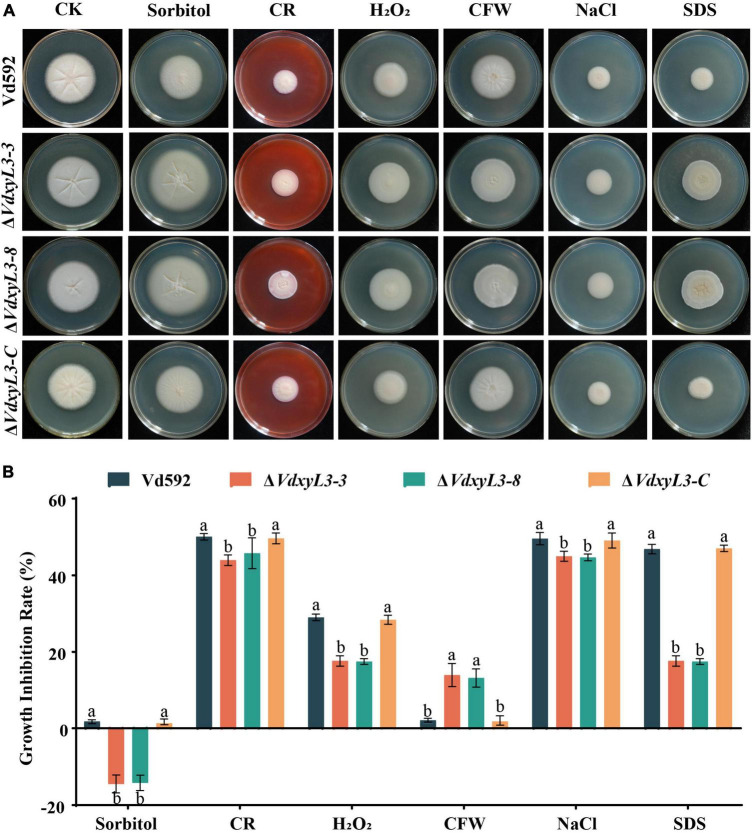
Impact of different osmotic stresses on colony morphology of different *V. dahliae* strains. **(A)** The four *V. dahliae* strains were cultured on PDA media supplemented with 1 M sorbitol, 0.02% Congo red (CR), 30% H_2_O_2_, 30 μg/mL calcofuor white (CFW), 1 M NaCl or 0.002% SDS. Colony morphology was observed and photographed after 15 days of incubation. **(B)** Colony diameter of all strains on different osmotic stresses. Error bars were based on three independent experiments. Data were statistically analyzed using IBM SPSS statistics 26.0. Significant differences between treatments were analyzed by one-way ANOVA using the Student-Newman-Keuls (SNK) test (different letters on error bars indicate significant difference at *P* ≤ 0.05).

To test the impact of *VdxyL3* deletion on the sensitivity of *V. dahliae* to different stresses, the four *V. dahliae* strains were cultured on PDA media with an addition of sorbitol, Congo red (CR), H_2_O_2_, calcofuor white (CFW), NaCl, or SDS. The *VdxyL3* deletion mutants grew faster on PDA media containing CR, sorbitol, CR, H_2_O_2_, NaCl and SDS (Figure 3A) and showed sigincantly lower growth inhibition rates compared to the Vd592 and complementation strain (Figure 3B). These results suggested that *VdxyL3* is involved in tolerance of *V. dahliae* to stress conditions.

### 3.4 Deletion of *VdxyL3* leads to enhanced carbon and nitrogen utilization capabilities of *V. dahliae*

**FIGURE 4:**
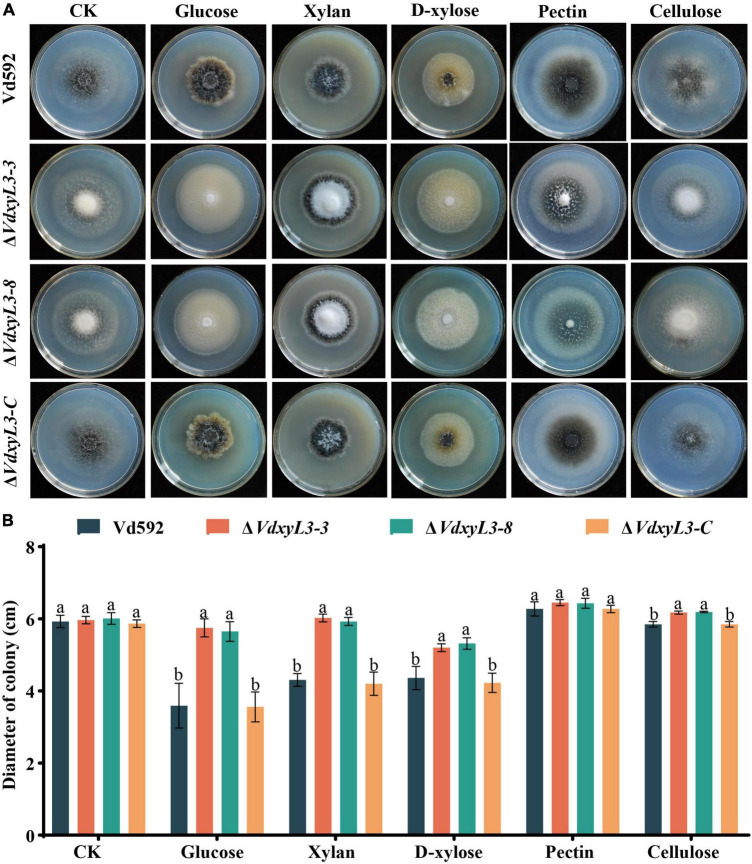
Colony morphology of different *V. dahliae* strains on different carbon sources. **(A)** Colony morphology of different *V. dahliae* strains on Czapek Dox medium supplemented with glucose, xylan, D-xylose, pectin or cellulose carbon sources. Images were taken at 15 days post incubation. **(B)** Colony diameter of all strains on different carbon sources. Error bars were based on three independent experiments. CK represents control. Data were statistically analyzed using IBM SPSS statistics 26.0. Significant differences between treatments were analyzed by one-way ANOVA using the Student-Newman-Keuls (SNK) test (different letters on error bars indicate significant difference at *P* ≤ 0.05).

**FIGURE 5:**
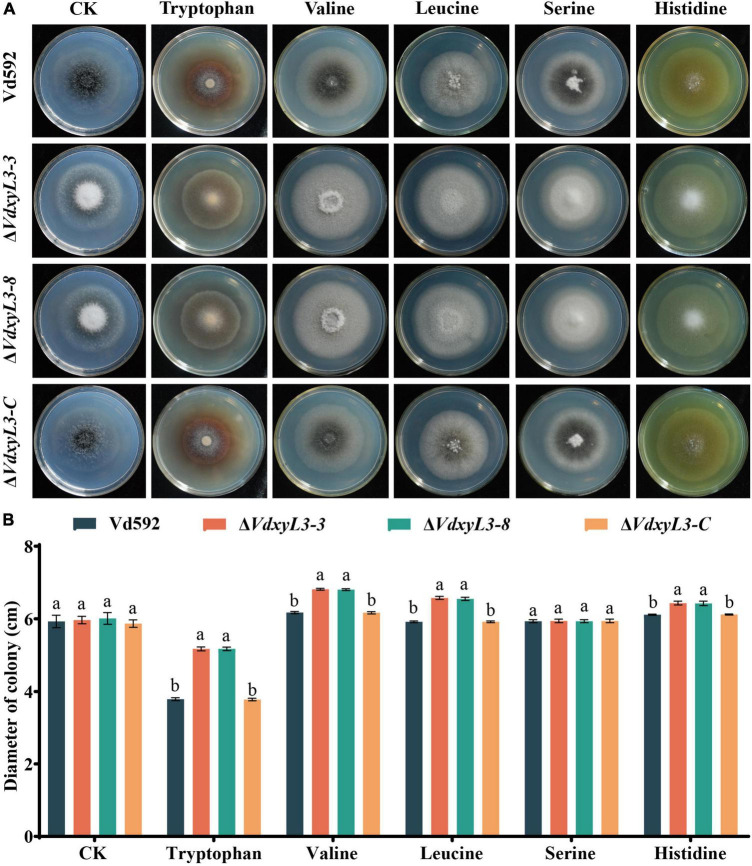
Colony morphology of different *V. dahliae* strains on different nitrogen sources. **(A)** Colony morphology of different V. *dahliae* strains on Czapek Dox medium supplemented with tryptophan, valine, leucine, serine or histidine nitrogen sources. Images were taken at 15 days post incubation. **(B)** Colony diameter of all strains on different nitrogen sources. Error bars were based on three independent experiments. CK represents control. Data were statistically analyzed using IBM SPSS statistics 26.0. Significant differences between treatments were analyzed by one-way ANOVA using the Student-Newman-Keuls (SNK) test (different letters on error bars indicate significant difference at *P* ≤ 0.05).

The increased colony growth rates of *VdxyL3* deletion mutants may be due to their capabilities to obtain more nutrients, such as sugars and amino acids. To verify this hypothesis, we conducted assays on the utilization capabilities of carbon and nitrogen sources of different strains. To test the capabilities of utilizing different carbon sources by the *VdxyL3* deletion mutants, the growth rates of the four strains were determined using five carbon sources, including xylan, D-xylose, glucose, pectin and cellulose. The colony diameters of the *VdxyL3* deletion mutants were obviously larger than that of the wild-type strain and the complementation strain on glucose, xylan, D-xylose and cellulose culture media (Figures 4A, B). To test the capabilities of utilizing different nitrogen sources by the *VdxyL3* deletion mutants, the growth rates of the four strains were determined using five nitrogen sources, including tryptophan, valine, leucine, serine and histidine. The colony diameters of the deletion mutants were obviously larger than that of the wild-type and the complementation strain on tryptophan, valine, leucine and histidine culture media (Figures 5A, B). These results indicated that *VdxyL3* is involved in carbon and nitrogen utilization of *V. dahliae*.

### 3.5 Deletion of *VdxyL3* leads to enhanced pathogenicity of *V. dahliae*

**FIGURE 6:**
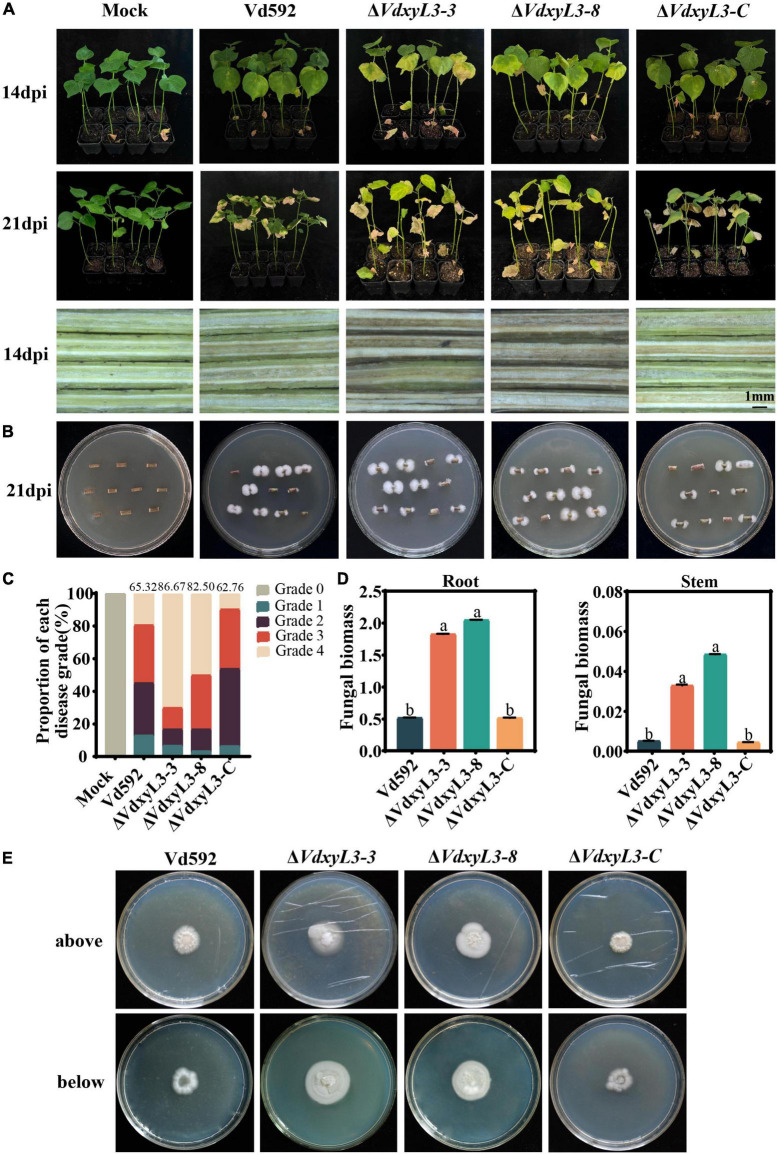
Deletion of *VdxyL3* led to increased pathogenicity of *V. dahliae*. **(A)** Disease symptoms of cotton plants (Xinluzao 7) infected with different *V. dahliae* strains at 14 and 21 dpi. **(B)** Fungal hyphae reisolated from cotton plants infected with different strains at 21 dpi. **(C)** Disease indices of cotton plants infected with different strains at 14 and 21 dpi. 0, 1, 2, 3, and 4 represent disease grade 0 to 4, representing 0, 0–25, 25–50, 50–75, and 75–100% wilted leaves, respectively. The bar chart represents the proportion of each disease grade, and the numbers on top of the column represent the DI value. **(D)** qRT-PCR assay of fungal biomass in infected roots and stems at 21dpi. **(E)** Deletion of the *VdxyL3* gene led to increased penetration capacity of *V. dahliae*. The indicated strains were grown on PDA medium overlaid with cellophane for 7 d and photographed (above). The plates were further incubated for 7 days after the removal of the cellophane layer and photographed (below). Data were statistically analyzed using IBM SPSS statistics 26.0. Significant differences between treatments were analyzed by one-way ANOVA using the Student-Newman-Keuls (SNK) test (different letters on different error bars indicate statistical difference at *p* < 0.05).

To determine the potential role of *VdxyL3* in pathogenicity of *V. dahliae*, cotton variety (Xinluzao 7) susceptible to *V. dahliae* was infected with Vd592 and the *VdxyL3* deletion mutant strains using the root irrigation method. Compared with Vd592 and the complementary strain (Δ*VdxyL3-C*), the *VdxyL3* deletion mutants showed significantly stronger pathogenicity to cotton (Figure 6A). Clearly increased disease symptoms, such as necrosis, wilting and vascular discoloration, as well as Disease Indices, were assessed in seedlings infected with the *VdxyL3* deletion mutants compared to those infected with Vd592 and complementary strain (Figures 6A, C). Correspondingly, fungal hyphae were isolated from plants infected with the deletion mutants at a higher rate of isolation than those of Vd592 and complementary strains (Figure 6B). Fungal biomass quantification by qRT-PCR revealed that treatments with the *VdxyL3* deletion mutants significantly increased the fungal biomass in seedling roots and stems compared to the treatments with the Vd592 and complementary strain of the fungus (Figure 6D).

To further characterize the effect of *VdxyL3* on pathogenicity, the penetration capability of hyphae of the wild-type and the mutant strains through the cellophane membrane was compared. At 7 days after removing cellophane membrane, on which different *V. dahliae* strains had been cultured for 7 days, the *VdxyL3* deletion mutants exhibited an obvious increase in colony growth from hyphae penetrating through the membrane compared to the wild-type and complementary strains (Figure 6E). These results suggest that the pathogenicity of the deletion mutants to cotton was significantly increased compared with that of the wild-type and complementary strains.

### 3.6 *VdxyL3.* is a secreted protein and fails to induce cell death in *N. benthaminana*

**FIGURE 7:**
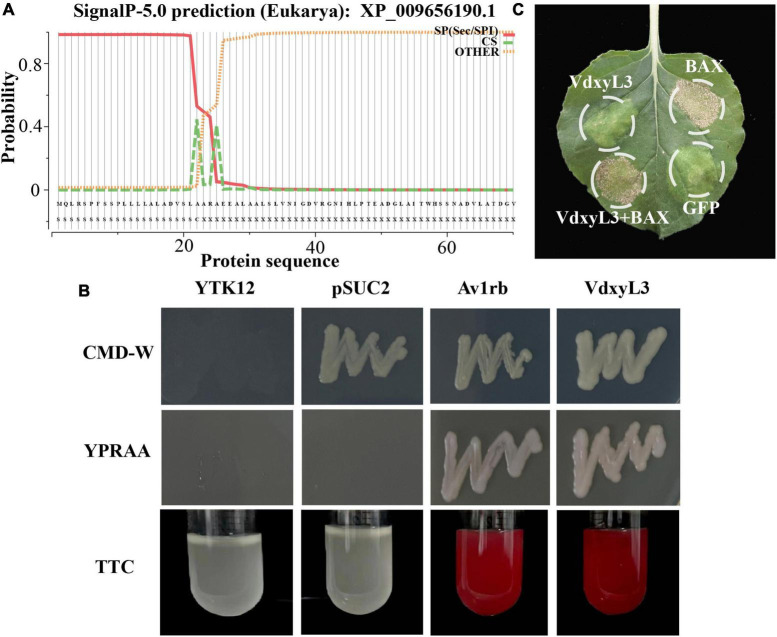
*VdxyL3* has secretory property and fails to induce cell death. **(A)** The predicted signal peptide site of the *VdxyL3* protein. **(B)** Functional validation of the signal peptide of *VdxyL3* by a yeast signal capture system. The sequence of the *VdxyL3* signal peptide was fused in-frame to the invertase sequence in the vector pSUC2, and then transformed into the yeast strain YTK12. The Avr1b with known function and empty PSUC2 were used as positive and negative control, respectively. **(C)** *VdxyL3* failed to induce cell death in tobacco leaves. Four-week-old tobacco plants were infiltrated with *Agrobacterium tumefaciens* carrying pYBA-1132: *VdxyL3* for transient expression. The pYBA-1132: GFP and PYBA-1132: BAX plasmid constructs served as negative and positive controls, respectively. Photographs were taken 7 days after infiltration.

*VdxyL3* putatively encodes a 522-amino-acid protein with a conserved glycoside hydrolase domain and has a predicted N-terminal signal peptide (SP, amino acids 1-22) (Figure 7A), indicating that *VdxyL3* could have secretory function. The secretory function of *VdxyL3* was tested based on the yeast signal trap assay system as previously reported (Tian et al., 2021). The SP of *VdxyL3* was fused to the pSUC2 vector to generate pSUC2-*VdxyL3*^SP^, which was transformed into yeast strain YTK12. YTK12 carrying pSUC2-*VdxyL3*^SP^ grew well in YPRAA medium with raffinose as the sole carbon source. The insoluble 2,3,5-triphenyltetrazolium chloride (TTC) was reduced to the insoluble red 1,3,5-triphenylformazan (TPF) (Figure 7B). The result confirmed that *VdxyL3* has secretory property and that it can be targeted to the extracellular environment under certain conditions. To test whether VdxyL3 protein is capable of inducing cell death in *N. benthamiana*, we used *Agrobacterium tumefaciens*-mediated transformation (agroinfiltration) to transiently express *VdxyL3* gene in *N. benthamiana* leaves. To do that, the coding sequence of *VdxyL3* including its secretion signal peptide was cloned into vector pYBA-1132. The pYBA-1132:GFP and PYBA-1132:BAX plasmid constructs served as negative and positive controls, respectively. *Agrobacterium* carrying *VdxyL3* or the control plasmids was injected into the leaves of 4-week-old *N. benthamina* plants. Transient expression of *VdxyL3* in *N. benthamiana* demonstrated that *VdxyL3* could not induce cell death in *N. benthamiana* at 7d after infiltration (Figure 7C) and failed to suppress cell death caused by BAX (Figure 7C).

### 3.7 RNA-seq analyses of the Δ*VdxyL3* strain of *V. dahliae*

To investigate the effect of *VdxyL3* deletion on gene expression in *V. dahliae* during infection process, transcriptomic analysis was conducted on cotton roots infected with the WT (Vd592) or Δ*VdxyL3* mutant strain. Clean sequencing data were mapped to the *V. dahliae* genome. The RNA-seq results were confirmed to be reliable by qRT-PCR using 12 randomly selected genes (Supplementary Table 2, Supplementary Figure 2). RNA-seq data revealed significant differences in expression of *V. dahliae* genes in cotton roots infected by WT or Δ*VdxyL3* mutant. In the roots infected by the WT strain, 6298 (3078 up-regulated and 3220 down-regulated) and 6408 (3172 up-regulated and 3236 down-regulated) differentially expressed genes (DEGs; *p* value ≤ 0.05, fold change ≥ 1.5) were identified in 36h vs 0h and 3d vs 0h comparisons, respectively (Supplementary Figure 3). In the roots infected by the Δ*VdxyL3* mutant, 6574 (3324 up-regulated and 3250 down-regulated) and 6348 (3010 up-regulated and 3338 down-regulated) DEGs were identified in 36h vs 0h and 3d vs 0h comparisons, respectively (Supplementary Figure 3).

**FIGURE 8:**
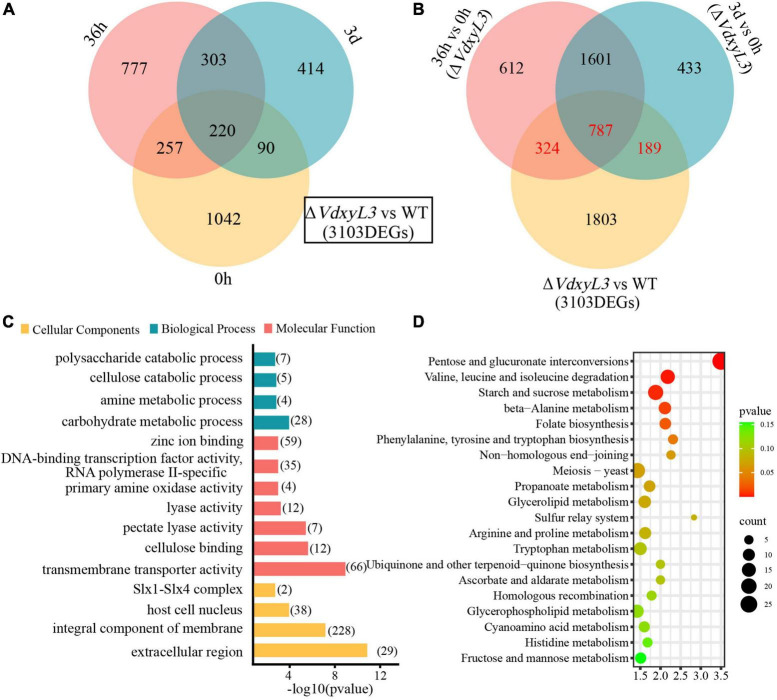
Identification of DEGs that may contribute to the enhanced pathogenicity of Δ*VdxyL3* mutant. **(A)** Venn diagram of up-regulated DEGs in Δ*VdxyL3* vs WT comparison at the three time points. **(B)** Venn diagram of up-regulated DEGs in 36 h vs. 0 h and 3 d vs 0 h comparisons of Δ*VdxyL3* and in Δ*VdxyL3* vs WT comparison. The numbers shown in red (324, 787, and 189) represent the DEGs contributing to the enhanced pathogenicity of the Δ*VdxyL3* mutant. **(C)** GO enrichment analysis of the putative pathogenesis-related DEGs. *X*-axis represents the -log10 (p value), and Y-axis represents the top 15 enriched GO terms. The number next to each horizontal bars represents the number of enriched DEGs in the corresponding GO term. **(D)** KEGG pathway analysis of the putative pathogenesis-related DEGs. X-axis represents the enrichment factor, and the *Y*-axis lists the top 20 pathways. The color of each bullet indicates the *p*-value and the size of the bullet reflects the number of enriched DEGs in the corresponding pathway.

Significant difference in gene expression was also evident between the WT and Δ*VdxyL3* mutant. A total of 4108 (1609 up-regulated and 2499 down-regulated), 6574 (3324 up-regulated and 3250 down-regulated), and 2669 (1027 up-regulated and 1642 down-regulated) DEGs were identified in the Δ*VdxyL3* vs WT comparison at 0 h, 36 h and 3 d post infection, respectively (Supplementary Figure 4). A total of 3103 up-regulated DEGs were identified in the Δ*VdxyL3* vs WT comparison at three time points (0, 36 h and 3 d) (Figure 8A). Of the 3103 DEGs up-regulated in Δ*VdxyL3*, the genes that were up-regulated in only 36 h vs 0 h (324) or 3 d vs 0 h (189), or both comparisons (787) were considered to contribute positively to the enhanced pathogenicity of the Δ*VdxyL3* mutant (Figure 8B).

To investigate the function of the 1300 DEGs up-regulated in Δ*VdxyL3*, GO (Gene Ontology) and KEGG (Kyoto Encyclopedia of Genes and Genomes) enrichment analyses were performed. The top 15 GO terms were listed in Figure 8C. In the cellular component category, extracellular region (GO:0005576) changed most significantly, followed by integral component of membrane (GO:0016021). In the molecular function category, most DEGs were significantly enriched in transmembrane transporter activity (GO:0022857), cellulose binding (GO:0030248), pectate lyase activity (GO:0030570) and lyase activity (GO:0016829). In biological process category, most DEGs were significantly enriched in carbohydrate metabolic process (GO:0005975) and polysaccharide catabolic process (GO:0000272). KEGG analysis showed that a high percentage of DEGs were involved in pentose and glucuronate interconversions (Ko00040), amino acids (valine, leucine, isoleucine and alanine) metabolism (Ko00280), and starch and sucrose metabolism pathways (Ko00500) (Figure 8D).

### 3.8 Deletion of *VdxyL3* affects the expression of potential pathogenesis-related genes

**FIGURE 9:**
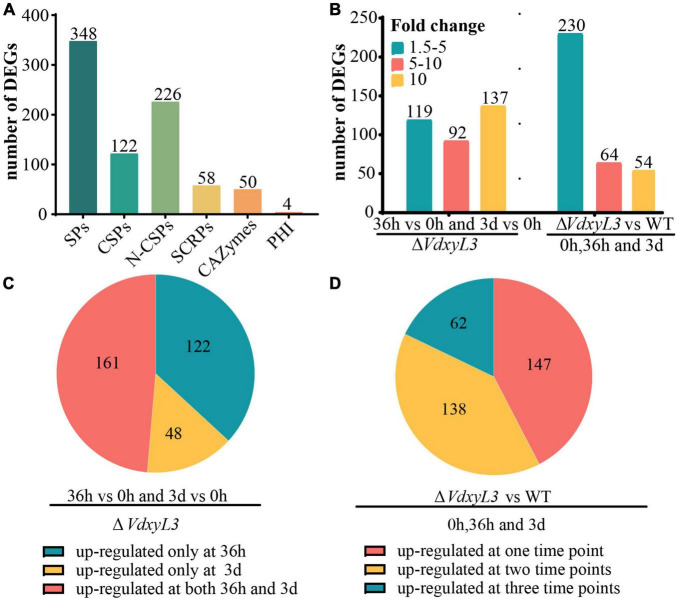
Deletion of *VdxyL3* affects the expression of potential pathogenesis-related genes. **(A)** The number of DEGs encoding secreted proteins. SPs, secreted proteins; CSPs, classical secreted proteins, N-CSPs, non-classical secreted proteins; SCRPs, small cysteine-rich proteins; CAZymes, carbohydrate-active enzymes; PHI, pathogen-host interaction-related proteins. **(B)** The number of DEGs with fold change ranging from 1.5 to 5, 5–10 and more than 10 in different comparisons. **(C)** Pie graph showing the number of up-regulated DEGs at 36 h or 3 d or both in the Δ*VdxyL3* mutant. **(D)** Pie graph showing the number of up-regulated DEGs in Δ*VdxyL3* vs WT comparison at one, two or all three time points.

Among the 1300 DEGs up-regulated in Δ*VdxyL3*, several genes were potentially involved in pathogenesis based on annotation, including 348 encoding secretory proteins (SPs) and 4 encoding pathogen-host interaction (PHI) homolog proteins (Figure 9A). Of the 348 SPs, 122 and 226 were classical secreted proteins (CSPs) and non-classical secreted proteins (N-CSPs), respectively (Figure 9A). The 122 classical secretory proteins included 50 carbohydrate-active enzymes (CAZymes) and 58 small cysteine rich proteins (SCRPs, <400 amino acids, ≧4 cysteine residues) (Figure 9A).

In the Δ*VdxyL3* mutant, the expression levels of 119 DEGs increased by 1.5–5 folds, 92 DEGs increased by 5–10 folds, and 137 DEGs increased by more than 10 folds (Figure 9B), with approximately half of the DEGs (161) up-regulated at both 36h and 3d two time points (Figure 9C). When compared to the WT strain, the Δ*VdxyL3* mutant had 230 DEGs increased their expression levels by 1.5–5 folds, 64 DEGs increased by 5–10 folds, and 54 DEGs increased by more than 10 folds (Figure 9B), with most DEGs (138 and 62) up-regulated at least at two time points (Figure 9D). In the two comparisons of Δ*VdxyL3*, more up-regulated DEGs were observed in 36h vs 0h than in 3d vs 0h (Supplementary Figure 5A), and between Δ*VdxyL3* vs WT, there was more up-regulated DEGs at 36h than the other two time points (Supplementary Figure 5B), suggesting that the 36h time point is crucial for the pathogenesis of *V. dahliae*.

### 3.9 Up-regulated DEGs encoding CAZymes may be responsible for the enhanced carbon utilization ability of Δ*VdxyL3* or act as effectors

**FIGURE 10:**
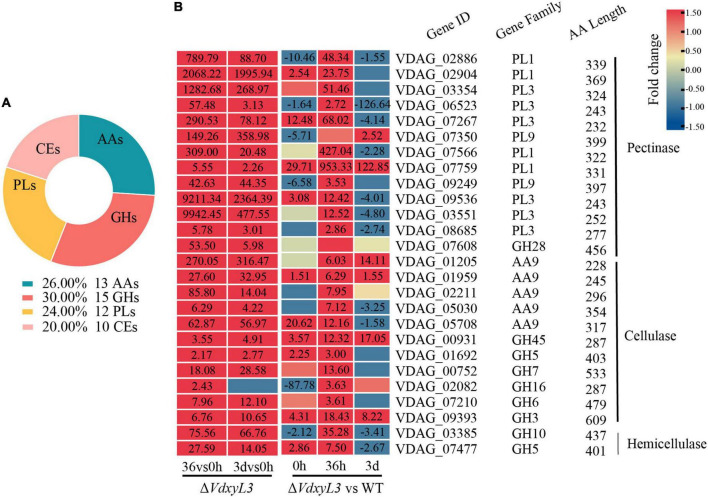
Catalog of DEGs encoding CAZymes and heatmap of DEGs encoding cell wall degrading enzymes. **(A)** Catalog of DEGs encoding CAZymes. GHs, glycoside hydrolases; PLs, polysaccharide lyases; AAs, auxiliary activities; CEs, carbohydrate esterases. **(B)** Heatmap showing the expression fold change of DEGs encoding cell wall degrading enzymes in different comparisons. The heatmap was generated based on the RNA-seq data. The numbers in heatmap are fold change, with those greater than or equal to 1.5 displayed.

Of the 50 secreted proteins annotated as CAZymes, 15 were glycoside hydrolases (GHs), 13 were auxiliary activity (AA), 10 were carbohydrate esterases (CEs), and 12 were polysaccharide lyase (PLs) (Figure 10A). Many of these CAZymes were involved in plant cell-wall degradation, including 13 pectinases, 11 cellulases and 2 hemicellulases (Figure 10B). The up-regulation of these CAZyme encoding genes might be responsible for the enhanced carbon utilization ability of Δ*VdxyL3* shown in Figure 3.

**FIGURE 11:**
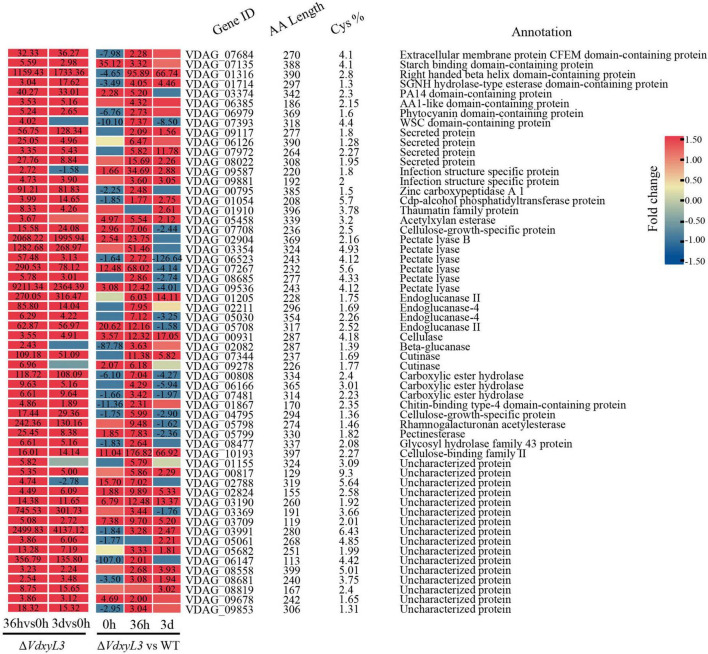
Heatmap showing the expression fold change of SCRP genes in different comparisons. The heatmap was generated based on the RNA-seq data. The numbers in heatmap represent fold change, with those greater than or equal to 1.5 displayed.

It was reported that several CWDEs and CFEM domain-containing extracellular membrane proteins can act as effectors during plant-pathogen interactions (Quoc and Chau, 2017; Wang et al., 2022; Su et al., 2024). Of the 58 DEGs annotated as SCRPs, several were plant cell wall degrading enzyme genes, including 6 pectate lyase genes, 4 endoglucanase genes, and 2 cutinase genes (Figure 11). One of the SCRPs (VDAG_07684) was a CFEM domain-containing extracellular membrane protein. Several other potential SCRPs were annotated as starch binding domain-containing protein, righthanded beta helix domain-containing protein, SGNH hydrolase-type esterase domain-containing protein, and PA14 domain-containing protein, whose function has not been reported yet, requiring further research.

## 4 Discussion

Xylosidases are crucial for the degradation of xylan in plant cell walls. Identifying xylosidase-encoding genes related to the pathogenicity of *V. dahliae* can provide a theoretical basis for the prevention and treatment of Verticillium wilt caused by *V. dahliae*. Xylosidase belongs to the glycoside hydrolase (GH) family, which is capable of hydrolyzing glycosidic bonds in sugar-containing compounds. According to the CAZyes database, the glycoside hydrolases were classified into 168 subfamilies (GH1-168) based on sequence similarity (Lombard et al., 2014). Xylosidases were separated into GH3, GH31, GH30, GH39, GH43, GH52, GH54, and GH120 subfamilies (Rohman et al., 2019). In this study, we identified 13 xylosidase genes from *V. dahliae*, which were grouped into GH3, GH13 or GH43 subfamily. Previous studies have shown that some GH subfamilies, such as GH11, GH12, GH61, whose members were required for the pathogenicity of pathogen (Brito et al., 2006; Ma et al., 2015; Chen et al., 2022). But the functional characterization of GH3, GH13 and GH43 members has not been reported yet. Among the 13 xylosidase gene, *VdxyL3* belonging to the GH43 subfamily was obviously induced by root exudates from cotton variety susceptible to *V. dahliae*, suggesting that it may be required for the infective process of *V. dahliae*.

Phytopathogenic fungi require the secretion of various hydrolytic enzymes to break down complex polysaccharides in the plant cell wall in order to invade the hosts and cause disease. Fungi produce different types of cell wall degrading enzymes (CWDEs) when infecting hosts. Many CWDE genes have been confirmed to be virulence factors, and their deletion mutants are significantly reduced in virulence. For example, the absence of *FoEG1*, *PsXEG1*, *VdPEL1*, *VdEG1* or *VdEG3* reduced the virulence of pathogen to cotton (Ma et al., 2015; Gui et al., 2017; Yang et al., 2018b; Zhang et al., 2021), suggesting that these genes contribute to the virulence of pathogen. In this study, the deletion of *VdxyL3* increased the virulence of *V. dahliae* to cotton, which is contrary to previous research findings, demonstrating the complexity of the pathogenic mechanism of *V. dahliae*. Although CWDEs are important for plant infection and colonization, not all fungal CWDEs have been conclusively shown to be involved in pathogenicity, probably due to some CWDE genes being less important or redundant (Apel et al., 1993; Gómez-Gómez et al., 2001; Sella et al., 2013). For example, a deletion mutant of the pectate lyase gene *PelA* does not show decreased virulence during infection (Blum, 2017).

Xylosidases are for degradation of xylan. Knockout of genes encoding xylosidase may lead to slower fungal growth, as deletion mutant strains are unable to obtain more nutrients through the degradation of xylan. Unexpectedly, the deletion of *VdxyL3* gene in this study resulted in increased colony growth rate, conidial production and mycelial growth in this study. The rapid growth of *VdxyL3* deletion mutant may be due to enhanced utilization capabilities of carbon and nitrogen sources, which were confirmed by our carbon and nitrogen sources assays (Figure 5). Comparative analysis of the transcriptomes of the wild-type and *ΔVdxyL3* strains during infective process revealed that *VdxyL3* deletion have an effect on the expression level of a large number of genes. Many CWDE genes were found to be up-regulated in Δ*VdxyL3* strain (Figure 10), and a high percentage of up-regulated DEGs were enriched in amino acids metabolism pathways (Figure 8D), which may be responsible for the enhanced utilization capabilities of carbon and Nitrogen sources, in terns lead to increased growth of Δ*VdxyL3* strain.

Enhanced fungal growth contributed to the enhanced pathogenicity of *V. dahliae* (Figure 6A). Comparative transcriptomic analysis also revealed up-regulation of 348 secreted protein encoding genes in *ΔVdxyL3*. It is notable that 58 of the 348 secreted protein encoding genes are genes encoding small cysteine rich proteins (SCRPs). It has been reported that SCRPs have low sequence similarity with known proteins, domains and motifs. Moreover, most fungal SCRPs exhibit significant genetic diversity among different species, making it difficult to predict the virulence and biochemical function based on sequence homology (Rep, 2005; Lo Presti et al., 2015; Tariqjaveed et al., 2021). So far, only a small number of SCRP genes in *V. dahliae* have been reported. Some SCRPs can suppress the plant immune responses, such as *VdSCP41* and *VdCP1* (Zhang et al., 2017; Qin et al., 2018), while some can induce the plant immune responses, such as *VdSCP27*, *VdSCP113* and *VdSCP126* (Wang et al., 2020). The 58 SCRP genes identified in this study were up-regulated in *ΔVdxyL3* and might contribute to the enhanced pathogenicity of *ΔVdxyL3*, an observation needs to be confirmed by further research.

RNA-seq of *V. dahliae* cultured in liquid medium containing cotton root tissue or treated by root extrudates has often been used in studies of the *V. dahliae*-cotton interaction (Zhang et al., 2018, 2020; Qiu et al., 2024). In this study, the root tissue carrying *V. dahliae* was taken from the intact plant and used for RNA-seq, which can more accurately reflect the changes in gene expression levels during the infection process of *V. dahliae*. The results presented in this study provide an experimental basis for future studies on the interaction between *V. dahliae* and cotton, and the pathogenic mechanism of the fungus.

## Data availability statement

The datasets presented in this study can be found in online repositories. The names of the repository/repositories and accession number(s) can be found in this article/Supplementary material.

## Author contributions

YTL: Conceptualization, Investigation, Writing – original draft, Writing – review and editing. SS: Investigation, Writing – original draft. BC: Investigation, Writing – original draft. YZ: Writing – original draft. TS: Investigation, Writing – original draft. XM: Writing – original draft. YL: Project administration, Writing – original draft, Writing – review and editing. JS: Conceptualization, Supervision, Writing – review and editing, Project administration. XZ: Conceptualization, Supervision, Writing – review and editing.

## Footnotes

1
https://www.ncbi.nlm.nih.gov/

2
http://smart.Emblheidelberg.de/

3
http://www.cbs.dtu.dk/services/TMHMM/

4
http://www.cbs.dtu.dk/services/SignalP/index.php

5
www.biocloud.net

6
http://www.bioinformatics.com.cn/
