# Supplementary material for: Deleting an xylosidase-encoding gene VdxyL3 increases growth and pathogenicity of Verticillium dahlia
Source: Front Microbiol. 2024 Jul 22;15:1428780. doi: 10.3389/fmicb.2024.1428780 (PMC11298495; doi:10.3389/fmicb.2024.1428780)
Supplement: Supplementary file 1 [file Data_Sheet_1.ZIP › Supplementary_Material (1).docx]

Supplementary Material

Deleting an Xylosidase-encoding gene *VdxyL3* Increases Growth and Pathogenicity of Verticillium dahlia

First Author* Yongtai Li, Co-Author, Shenglong Song

*** Correspondence:**

Jie Sun [sunjie@shzu.edu.cn](mailto:sunjie@shzu.edu.cn)

Xinyu Zhang zhxy@shzu.edu.cn

# Supplementary Figures and Tables

## Supplementary Tables

**Table S1 Primer sequences used in this study**

| Gene name | Primer name | Primer sequence（5′–3′） |
| --- | --- | --- |
| *VdxyL3* | *VdxyL3*-OFR-F | ATGACGCTTCTTTCCAAGTCG |
|  | *VdxyL3*-OFR-R | TTAATACCCCCTCAACTTGGC |
|  | *VdxyL3*-Flank-5F | GGTGGCGGCCGCTCTAGACTACCTACCTTGGGTACTTACCTTA |
|  | *VdxyL3*-Flank-5R | AAAATGCTCCTTCAAGGTTGACGAGCACGATGCCCAAACT |
|  | *VdxyL3*-Flank-3F | GGTTCGCAAAGATAATTGTCCAAGCCCAACGACGCTGTTCGCC |
|  | *VdxyL3*-Flank-3R | GACGGTATCGATAAGCTTTTAATACCCCCTCAACTTGGCAAAC |
|  | *VdxyL3*-HPH-F | TCGTGCTCGTCAACCTTGAAGGAGCATTTTTGGGCTTGGCTGG |
|  | *VdxyL3*-HPH-R | GTTGGGCTTGGACAATTATCTTTGCGAACCCAGGGCTGGTG |
|  | *Promotor-F* | ACGGCCAGTGCCAAGCTTCTATTTTCACGTCTGCTCGAA |
|  | *Promotor-R* | ATTCACTAGTCAGGATCCACAATTTGCAGGTCGTTTGTA |
|  | *Test*-*VdxyL3*-*F1* | AAGCTCATGCGACCTAGCACACGCACC |
|  | *Test*-*VdxyL3*-*R1* | GAAGTAACCGGACAGCGTGTCATTCCA |
|  | *Test*-*VdxyL3*-*F2* | AAGCTCATGCGACCTAGCACACGCACC |
|  | *Test*-*VdxyL3*-*R2* | GATAAACATAACGATCTTTGTAGAAACC |
|  | *Test*-*VdxyL3*-*F3* | CGAGGACTGCCCCGAAGTCCGGCAC |
|  | *Test*-*VdxyL3*-*R3* | GAAGTAACCGGACAGCGTGTCATTCCA |
|  | *Test*-Hyg-F | AAGGTGAGTCCTCCAACCA |
|  | *Test*-Hyg-*R* | GAAGCACGAATCCTCTTGTT |
|  | *VdxyL3*-F | GGAATTCCTGGGCCTCTTCGCTCTATG |
|  | *VdxyL3*-R | GGGTACCACAGCACTGAGACCAGCATC |
|  | *VdPT1*-qF | CGGAAACATTCACCTCCCCA |
|  | *VdPT1*-qR | GGCGTAAGCCTCGAAAGCAT |
| *Tubulin* | *Tubulin*-F | TTTCCAGATCACCCACTCC |
|  | *Tubulin*-R | ACGACCGAGAAGGTAGCC |
| *Ve-ITS1* | *Ve-ITS1*-F | AAAGTTTTAATGGTTCGCTAAGA |
| *ST-VE1* | *ST-VE1*-R | CTTGGTCATTTAGAGGAAGTAA |
| *GhUBQ7* | *UBQ7*-F | GAAGGCATTCCACCTGACCAAC |
|  | *UBQ7*-R | CTTGACCTTCTTCTTCTTGTGCTTG |
| pSUC2- *VdxyL3* (SP) | pSUC2- *VdxyL3* (SP)-F | GGAATTTTAATTAAGAATTCATGCAGCTCCGCTCGC |
|  | pSUC2- *VdxyL3* (SP)-R | ACTATAGGGAGAACCTCGAGTCCATCTACCCCCTCCAACTGA |
| PYBA-1132: *VdxyL3* | PYBA-1132: *VdxyL3-F* | TAGAACTAGTGGATCATGCAGCTCCGCTCGC |
|  | PYBA-1132: *VdxyL3-R* | CGGTATCGATAAGCTTCCATCTACCCCCTCCAACTGA |

Note:The underlined part is the restriction sites. *Eco*RI:GAATTC, *Kpn*I: GGTACC

**Table S2 Primer list for gene validation by RNA-Seq analysis**

| Primer name | Primer sequence（5′–3′） |
| --- | --- |
| VDAG_00931-qF | ATGCACGCCAACCTCCTGA |
| VDAG_00931-qR | GTCGTTGATGTCGCAGGTCG |
| VDAG_01205-qF | CGCACTGTATGTAGCGATGGATCA |
| VDAG_01205-qR | GTAACATGTCAACTGCTCGCTGG |
| VDAG_01316-qF | ATCATCGGACCCAAAGTCAAGC |
| VDAG_01316-qR | GACGACCGTGTTATGCTTGAC |
| VDAG_02904-qF | CTGACATGGACTACAGGATGGAC |
| VDAG_02904-qR | CTTGAAGGAGGAGGCGAACT |
| VDAG_03190-qF | ATGCTGACGAATGTTGCCCTC |
| VDAG_03190-qR | TCATTGTTAAAGTACCTGCTGGCG |
| VDAG_03354-qF | CCTCAATGCCAACACAAACAGT |
| VDAG_03354-qF | GTGTAGTGGCACGAGCACTC |
| VDAG_03551-qF | CAAGGTGATTTGGGAGAGAGG |
| VDAG_03551-qR | TACCTTCGGCCTGCTCATTAC |
| VDAG_03991-qF | TCTTTTCTCTCGCTCGTCGACA |
| VDAG_03991-qR | CGTCTCATCAATACACGTCTCGG |
| VDAG_07759-qF | ACCATCTCTGAGGCTGCTAC |
| VDAG_07759-qR | ATGATGCCCTTCACAACAACAAT |
| VDAG_09536-qF | CTCTCTGTCTCAGCCTTCCAC |
| VDAG_09536 -qR | GCGTCTTTTGCCATGACACTA |
| VDAG_09881 -qF | GAACATCAAGCTCCAAAGCCT |
| VDAG_09881 -qR | CATGAGGTCCTCCGACAGC |
| VDAG_10193 -qF | TGGGAAGGATGCCACCAGTT |
| VDAG_10193 -qR | TGAGCTGGTCCCAGCCATAG |

## Supplementary Figures

**Figure S1**

**Supplementary Figure S1.** Cloning and gene expression of *VdxyL3*.

**(A)** PCR confirmation of the Δ*VdxyL3* mutant strains. **(B)** PCR confirmation of the Δ*VdxyL3-C* and Δ*VdxyL3* strain. **(C)** The relative expression level of *VdxyL3* in *VdxyL3* and Δ*VdxyL3-C* strains.

**Figure S2**

**Supplementary Figure S2.** Confirmation of RNA-seq via qRT-PCR analysis. The expression of twelve selected DEGs from the RNA-seq analysis were measured by qRT-PCR in Δ*VdxyL3*-0h, Δ*VdxyL3*-36h, Δ*VdxyL3*-3d. The histograms were plotted using data obtained by qRT-PCR and the corresponded line chart was plotted by FPKM values from the RNA-seq analysis. Each bar represents the mean value with standard errors of three independent experiments.

**Figure S3**

**
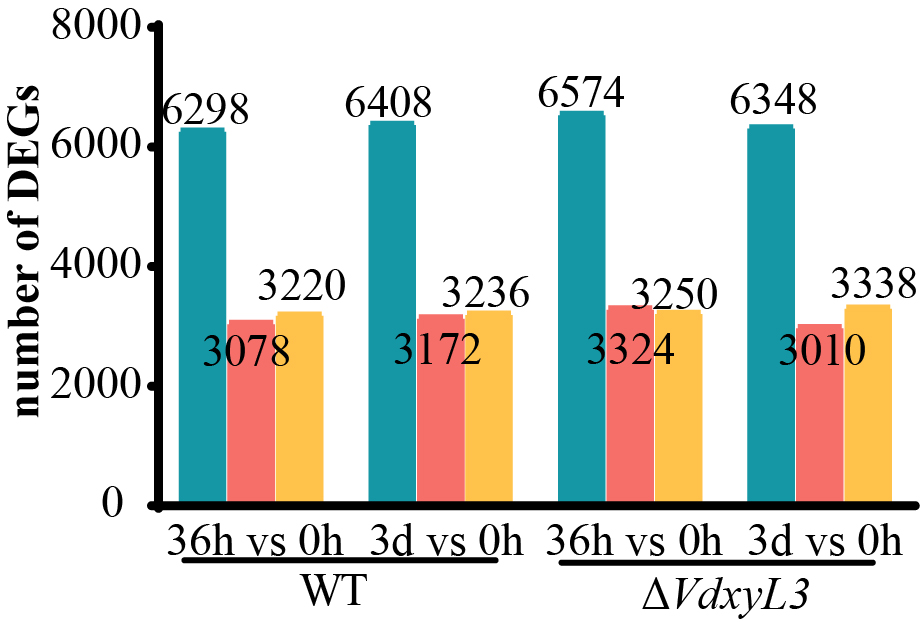
**

**Supplementary Figure S3.** Number of DEGs identified in 36h vs 0h and 3d vs 0h comparisons of Δ*VdxyL3* and WT strains. The x-axis represents different comparisons and the y-axis represents the number of DEGs identified.

**Figure S4**

**
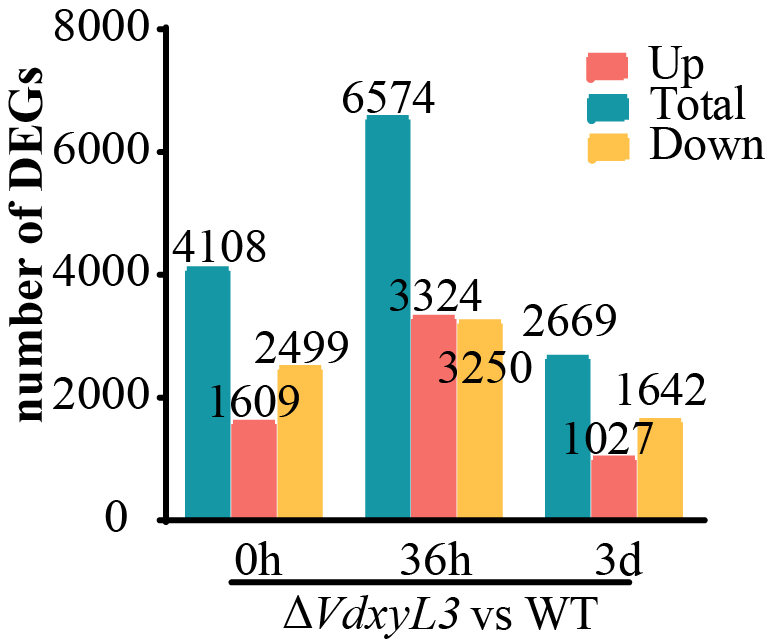
**

**Supplementary Figure S4.** Number of DEGs identified in Δ*VdxyL3* vs WT comparison at the three time points. The x-axis represents different comparisons and the y-axis represents the number of DEGs identified.

**Figure S5**

**
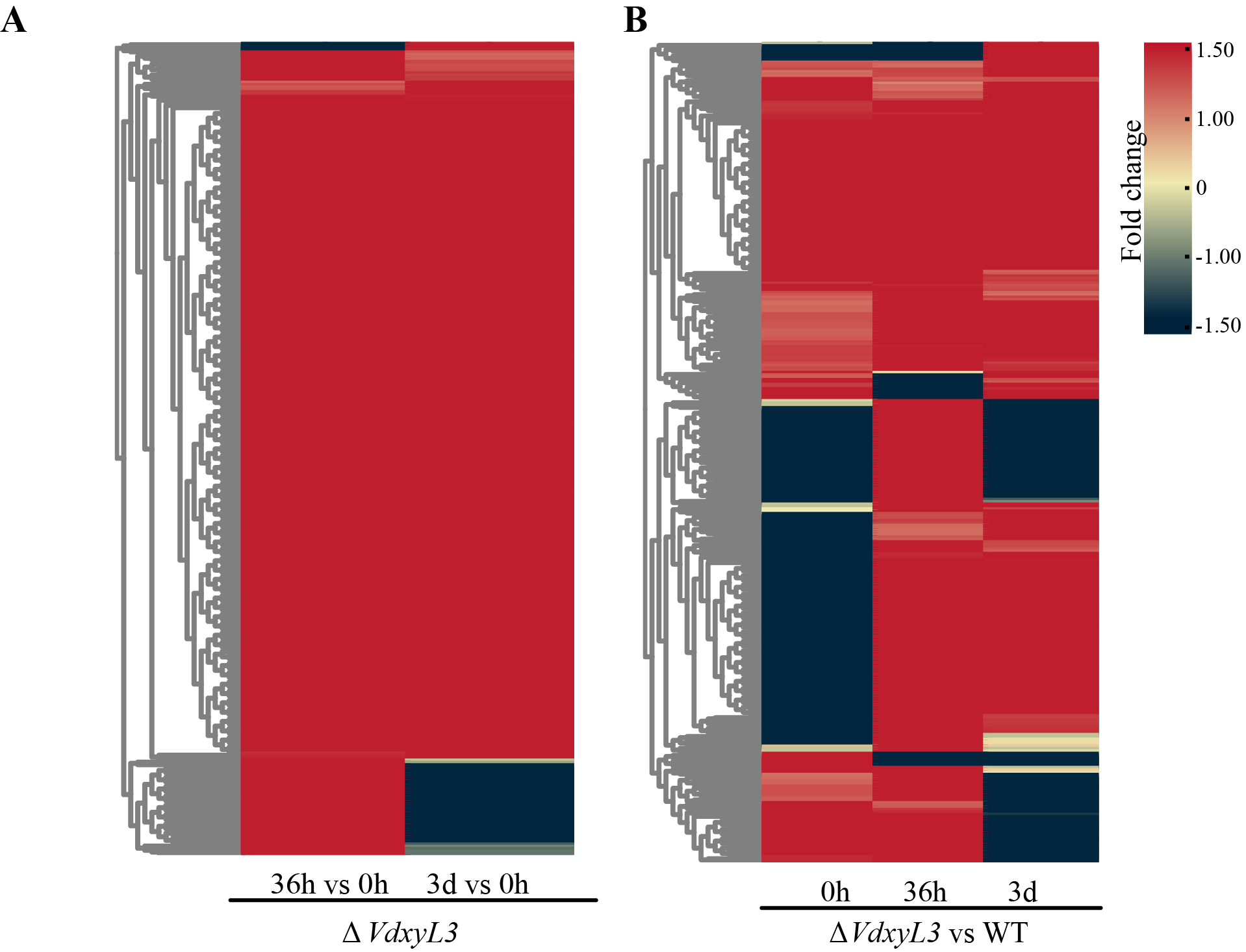
**

**Supplementary Figure S5. (A, B)** Hierarchical cluster analysis of DEGs encoding secreted proteins. The heatmaps were generated based on the expression fold change of the genes in different comparisons.
